# Dynamic Proximity Networks of Myosin-19 (Myo19) and its Mitochondrial Receptors Miro2 and Metaxin-3

**DOI:** 10.1016/j.mcpro.2026.101582

**Published:** 2026-05-06

**Authors:** Aya Attia, Jürgen Eirich, Ulrike Honnert, Petra Nikolaus, Birgit Lohmann, Iris Finkemeier, Martin Bähler

**Affiliations:** 1 Institute of Integrative Cell Biology and Physiology, University of Münster, Münster, Germany; 2 Medical Molecular Genetics Department, National Research Centre, Dokki, Cairo, Egypt; 3 Institute of Biology and Biotechnology of Plants, University of Münster, Münster, Germany

**Keywords:** TurboID proximity labelling, OMM, Myo19, Miro2, MTX3, mitochondrial dynamics, MIB/MICOS, ERMCS, myosin, actin, mitochondria

## Abstract

Myosin-19 (Myo19) plays a crucial role in mitochondrial dynamics, cristae organization and ER-mitochondria contact sites. It regulates cytokinesis and inheritance of mitochondria to daughter cells. To better understand the dynamic molecular network of Myo19 during the cell cycle, we determined the *in vivo* proximity protein interaction networks of Myo19 in interphase and prometaphase using proximity-based TurboID biotinylation followed by mass spectrometry. We further determined the proximity networks of its known mitochondrial binding partners Miro2 and metaxin-3. The outer mitochondrial membrane protein Miro2 not only binds but also stabilizes Myo19. This interaction depends on the nucleotide state of the N-terminal GTPase domain of Miro2. Therefore, we analysed both the proximity networks of Miro2 and its GTP-binding mutant Miro2 T18N. We were able to show a differential association of Myo19 during the cell cycle with functional protein clusters and a participation of Myo19 in mitochondrial trafficking, ER-mitochondria contact sites and mitochondria intermembrane space bridging complex/cristae organizing system. The proximity network of Myo19 showed more overlap with Miro2 than metaxin-3. Abolishing GTP-binding to the N-terminal GTPase domain of Miro2 reduced the number of proteins in proximity of Miro2 considerably. In conclusion, we discovered a comprehensive dynamic *in vivo* protein proximity network of Myo19 and its mitochondrial receptors Miro2 and metaxin-3.

## Highlights

• The protein proximity network of Myo19 undergoes cell cycle dependent remodeling.
• Myo19 classifies as an ERMCS protein.
• Myo19 is in close proximity of MIB/MICOS.
• The Miro2 protein proximity network is regulated by the nucleotide state of the N-terminal GTPase domain.
• Myo19 and Miro2 protein proximity networks show more overlap with each other than with Metaxin 3.

## Graphical Abstract

**Figure:**
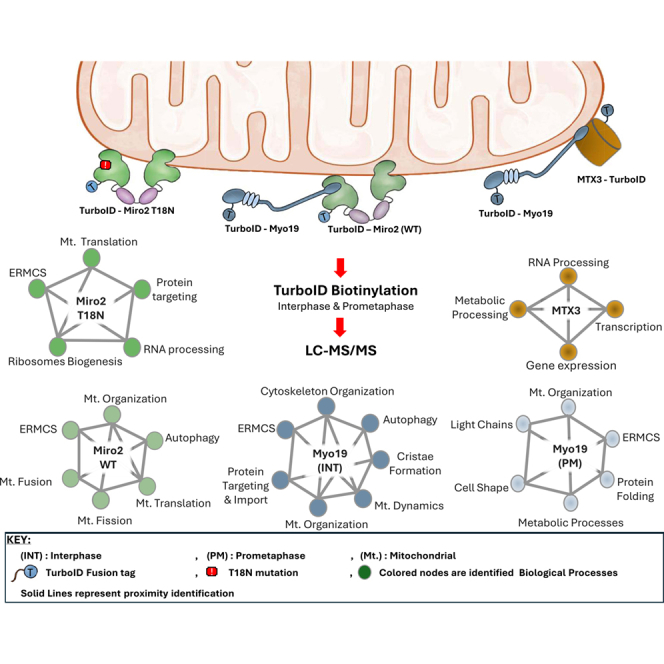

## In Brief

We report the protein proximity network of the mitochondrial actin-based motor Myo19 in interphase and at prometaphase. Further, we report the protein proximity networks of its mitochondrial receptors Miro2 and metaxin-3. Additionally, we determined the protein proximity network of Miro2 with a dominant negative point mutation in its N-terminal GTPase domain.

Mitochondria are highly dynamic, semi-autonomous organelles of eukaryotic cells. In mammals, mitochondrial genomes are relatively small and highly conserved (1, 2). Mitochondria and their genome are surrounded by two membranes, an inner mitochondrial membrane (IMM) and an outer mitochondrial membrane (OMM). The inner membrane encloses the matrix compartment and partly runs parallel to the outer membrane that encloses the intermembrane space. However, the inner membrane has many invaginations to the matrix forming separate sheets and tubes called cristae. The cristae membrane contains the enzymes that generate the cellular fuel ATP by Oxidative Phosphorylation. Mitochondria biogenesis relies on translocation of proteins, encoded by the nuclear DNA, from the cytosol across or into the membranes. Additionally, mitochondria also exchange components with other organelles, for example the endoplasmic reticulum, *via* specialized membrane contact sites (3). Further, mitochondria can fuse with each other to form extensive networks. On the other hand, mitochondria can undergo fission and become fragmented. Hence, mitochondria can decrease or increase in number and size. During the cell cycle mitochondria fragment at mitosis and segregate passively or actively to the two daughter cells during cytokinesis (4). Their morphology and distribution are determined by the cytoskeleton. Mitochondria can be anchored or transported along microtubules and actin filaments. Directed force and transport along the cytoskeleton is mediated by cytoskeletal polymerization and motor proteins. The outer mitochondrial membrane proteins Miro1 (RhoT1) and Miro2 (RhoT2) serve as receptors for the microtubule-based motor proteins kinesin KIF5 and dynein (5, 6, 7). The proteins TRAK1 and TRAK2 mediate as adaptors the interaction of the motor proteins with the Miro1/2 mitochondrial receptor proteins (6, 7, 8, 9, 10, 11, 12). Interestingly, the actin-based mitochondrial motor Myo19 binds directly to Miro1/2 and competes for binding with the adaptor proteins of the microtubule-based motors (12). In mammalian cells, directed transport of mitochondria is mostly microtubule-based (13). Currently, it is not known how actin-based and microtubule-based force production are coordinated by the shared receptors Miro1/2. They consist of a N-terminal Rho subfamily related GTPase domain that is followed by two Ca^2+^-binding domains, a second GTPase domain and a C-terminal transmembrane anchor (14, 15). Deletion of Miro led to a concomitant downregulation of Myo19, but not vice versa (12, 16). Deletion of Myo19 affects the distribution and morphology of the mitochondria (17). Specifically, during mitosis causes the lack of Myo19 an asymmetric accumulation of mitochondria at spindle poles (17, 18). During mitosis actin cables take over an important role in scaffolding mitochondria and the ER in the subcortical region (19). Myo19 appears to tether mitochondria and associated ER to actin cables as the depolymerization of actin filaments led to a comparable asymmetric accumulation of mitochondria at the spindle poles and a collapse of the ER (19). Furthermore, during mitosis fusion of mitochondria is not interrupted in cells lacking Myo19, possibly because mitochondria are no longer tethered to the actin cables which impedes their motility and fusion. This prevents proper mitochondria fragmentation which appears to induce stochastic failures of cytokinesis (17, 18, 19). Furthermore, both Myo19 and Miro deficiency reduces the number of mitochondria-ER membrane contact sites that mediate the transfer of Ca^2+^ and lipids between the two organelles. Not only are activities related to the OMM affected, but also the folding and architecture of the IMM (20, 21, 22). Miro and Myo19 are linked to the mitochondrial contact site and cristae organizing system (MICOS) (20, 21, 23) The connection with MICOS ensures that force is not only applied to the OMM but is simultaneously transmitted to the IMM.

It is still poorly understood how Myo19 affects mitochondria distribution and fusion during mitosis and how it regulates the number of ER-mitochondria contact sites (ERMCS) and cristae organization. Therefore, we set out to identify proteins that *in vivo* are in proximity of Myo19 in interphase and prometaphase. In this study, we also included the proteins Miro2 and metaxin-3 (MTX3) which had been identified as mitochondrial interaction partners of Myo19 previously (12, 24). We mapped the local environment of these proteins in living cells by proximity labeling (25). To this end, the proteins of interest were fused to an engineered biotin ligase, specifically TurboID (26). The expressed fusion proteins served as “baits”. They form short lived reactive biotin intermediates that covalently label solvent exposed lysine residues of “prey” proteins in the immediate vicinity of about 10 to 20 nm radius (26). These biotinylated proteins are affinity-purified under denaturing conditions and analyzed by mass spectrometry. We report here the identification of proteins that are in the proximity of Myo19 during interphase and prometaphase and compare them with proteins in proximity of Miro2 in the GTP-bound and nucleotide-free form of the N-terminal GTPase domain. Additionally, to define a more comprehensive proximity network of Myo19 on the outer mitochondrial membrane, we determined proteins located in proximity of its putative binding partner metaxin-3. The determined *in vivo* proximity networks of Myo19, Miro2 and metaxin-3 provide a rich resource for elucidating the molecular mechanisms of their actions in mitochondrial biology.

## Experimental Procedures

### Experimental Design and Statistical Rationale

TurboID proximity-labeling experiments were performed using four independent biological replicates, defined as separate cell cultures processed independently through biotin labeling, affinity purification, and LC–MS/MS analysis. This sample size was chosen to provide sufficient statistical power while remaining consistent with established TurboID-based quantitative proteomics studies.

For each bait, a cytosolic TurboID-NES control was processed in parallel under identical conditions to control for nonspecific cytosolic biotinylation. No technical or process replicates were acquired. Each biological replicate was analyzed by a single LC–MS/MS run.

Statistical analysis was performed on log_2_-transformed label-free quantification (LFQ) intensities using the limma framework, with multiple testing corrections by false discovery rate (FDR). Proteins with log_2_ fold change ≥1 and FDR-adjusted *p*-value ≤0.05 were considered significantly enriched.

### Antibodies

The primary antibodies used in this study included anti-β-actin (mouse, 1 μg/ml for WB, catalog number AC-15/A1978 from Sigma-Aldrich), anti-Myo19 (rabbit anti-human, 0.176 μg/ml for WB, catalog number ab174286 from Abcam), anti-VDAC1 (mouse, 1 μg/ml for WB, catalog number ab14734 from Abcam), and anti-V5 Tag (mouse, 1:5000 for WB and 1:1000 for IF, catalog number AB_2556564 from Thermo Fisher Scientific). Additionally, we used the following secondary antibodies: anti-mouse IgG-HRP (goat, 1:5000 for WB, catalog number 115-035-003 from Jackson ImmunoResearch), anti-rabbit IgG-HRP (goat, 1:5000 for WB, catalog number 111-035-003 from Jackson ImmunoResearch), anti-mouse-IgG-Alexa Fluor 488 (goat, 1:500 for IF, catalog number 115-545-003 from Jackson ImmunoResearch), and anti-mouse-IgG-Cy3 (goat, 1:500 for IF, catalog number 115-165-003 from Jackson ImmunoResearch). To detect biotinylated proteins, we applied streptavidin–HRP (1:3000 for WB, catalog number S911 from Thermo Fisher Scientific).

### Plasmids

The plasmids used in this study were generated as follows: V5-TurboID-Miro 2 was constructed by amplifying V5-TurboID from the V5-TurboID-NES-pcDNA3 template (Addgene ID 107169) (26) using PCR. The amplified product was then cloned into the mTag BFP2-Miro2 plasmid using BshTI and XhoI, replacing mTag BFP2 with V5-TurboID. The mTag BFP2-Miro2 plasmid was previously generated by cloning the full-length human Miro 2 sequence into the mTagBFP2-C1 vector using EcoRI and Eco72I restriction sites. The plasmid mTag-BFP2-C1 was constructed by amplifying mTag-BFP2 from mTagBFP2-TOMM20-N-10 (Addgene ID 55328) (27) and subcloning it into the pEGFP-C1 vector using Bgl II and BshTI, replacing GFP with mTagBFP2. The V5-TurboID-Miro2 T18N plasmid was created by excising an 833 bp fragment from Miro 2 T18N (12) and replacing the corresponding fragment in the V5-TurboID-Miro 2 plasmid using XhoI and Eco72I. Plasmid V5-TurboID-Myo 19 was similarly constructed by amplifying the sequence coding for V5-TurboID from V5-TurboID-NES-pcDNA3 (Addgene ID 107169) (26). This fragment was subcloned with BshTI/BglII into plasmid GFP-Myo 19, replacing GFP with V5 TurboID. The V5-TurboID-Myo19 pIREShyg plasmid was generated by ligating a 323 bp Eco47III/SalI fragment and a 3633 bp SalI/XbaI fragment from V5-TurboID-Myo19 into the SmaI/NheI-digested pIREShyg Linker vector. The pIREShyg Linker, derived from the Clontech pIREShyg vector, was modified by introducing additional restriction sites into the multiple cloning site using a synthetic linker (28). Plasmid V5-TurboID-NES pIREShyg was constructed by ligating a 323 bp Eco 47III/Sal I fragment from V5-TurboID-Myo 19 and a 756 bp SalI/XbaI fragment from V5-TurboID-NES-pcDNA3 (Addgene ID 107169) (26) into SmaI/NheI-digested pIREShyg Linker.

To construct the pmCherry-N1 expression vector, a modified version of pEGFP-N1 (Clontech) was generated by replacing the EGFP coding sequence with the mCherry fluorescent protein sequence. First, the mCherry gene was amplified by PCR using primers MB1205F and MB1206R, with the plasmid pRSET-B-mCherry (a generous gift from Dr Roger Y. Tsien) as the DNA template. The resulting 746-bp PCR product was digested with BshTI and NotI, producing fragments of 726 bp, 14 bp, and 5 bp. Separately, the pEGFP-N1 plasmid was digested with the same enzymes (BshTI and NotI), yielding fragments of 3998 bp and 735 bp. The 726-bp mCherry fragment was then ligated to the 3998-bp pEGFP-N1 backbone, replacing the EGFP sequence and creating the pmCherry-N1 vector.

The Metaxin3-pmCherry-N1 plasmid was generated by amplifying the full-length human MTX3 cDNA from clone BC160186 (BioCat, Troy) and subcloning it into the pmCherry-N1 vector using XhoI and BamHI restriction sites. Plasmid Metaxin3-V5-TurboID was created by amplifying V5-TurboID from V5-TurboID-NES-pcDNA3 (Addgene ID 107169) (26) and subcloning it into the Metaxin3-pmCherry-N1 plasmid with BamHI/NotI, replacing mCherry with V5-TurboID.

### Culturing Eukaryotic Cell Lines

HEK Myo19 KO cell lines were generated using CRISPR/Cas9-mediated genome editing as described by Majstrowicz *et al*. (17). Routine cell culture for HEK and HeLa cells were performed in T75 cell culture flasks using complete DMEM, which contained 3.7 g/L NaHCO_3_, 0.58 g/L L-glutamine, 4.5 g/L D-glucose, and phenol red, supplemented with 100 U/ml penicillin, 100 μg/ml streptomycin (Pan Biotech), and 10% (v/v) heat-inactivated FCS (Biochrom). All cell lines were maintained at 37 °C with 5% CO2 and 95% humidity. Passaging was conducted at a 1:10 dilution twice a week when cells reached 80 to 90% confluence. For passaging, cells were washed with pre-warmed 1× PBS and treated with Trypsin-EDTA (PAN-Biotech) for 2 to 5 min to ensure detachment. The reaction was halted by adding 10 ml of complete growth medium. The cells were then pelleted by centrifugation at 300 × *g* for 5 min, resuspended in fresh culture medium and reseeded.

### Generation of Stable Cell Lines

To establish stable TurboID cell lines, 1 × 10^5^ HeLa WT or HEK Myo19-deficient cells were seeded into each well of a 6-well plate. Transfection was performed on the following day using 2.5 μg of linearized plasmid DNA, 5 μl Lipofectamine LTX (Thermo Fisher Scientific #15338100) and 2.5 μl PLUS Reagent in 250 μl DMEM. After a 4-h incubation, the transfection complexes were removed, and cells were washed with PBS before being replenished with fresh complete DMEM. Forty-eight hours post-transfection, cells were re-plated at 60%, 30%, and 10% density in 150 mm cell culture dishes. Selective media containing 600 μg/ml G418 was used for HeLa TurboID-Miro2, TurboID-Miro2 T18N, MTX3-TurboID, and TurboID-NES cells, while 100 μg/ml Hygromycin was used for HEK Myo19-deficient TurboID-Myo19 and TurboID-NES cells. The selective medium was refreshed every third day, and stably transfected cells were identified after 2 weeks.

Individual cell colonies were isolated using metal rings and trypsinized for further processing. Detached cells were split and one portion was prepared for Western blot analysis and the other expanded as a stable cell line. Stable cell lines were screened for moderate TurboID fusion expression, as assessed by V5 immunoblotting and streptavidin-HRP labeling patterns. Cell clones were aliquoted, cryopreserved and stored in liquid nitrogen for long-term use.

### Maintenance of Stable Cell Lines

Stable HeLa and HEK Myo19-deficient cell lines were maintained in complete DMEM supplemented with the appropriate antibiotics. HeLa TurboID-Miro2, TurboID-Miro2 T18N, MTX3-TurboID, and TurboID-NES cells were cultivated with 600 μg/ml G418, while HEK Myo19-deficient TurboID-Myo19 and TurboID-NES cells were cultured with 100 μg/ml Hygromycin to ensure stable expression.

### Cell Synchronization

To synchronize cells at the prometaphase stage of the cell cycle, a drug treatment protocol was employed. Initially, cells were treated with 2 mM thymidine for 16 h (Santa Cruz #296542A), prepared as a 100 mM stock solution in dH_2_O and stored at −20 °C. Following this treatment, cells were released into fresh complete medium for 8 h to allow progression through the cell cycle. Subsequently, a 20-h incubation with 5 μM S-trityl-L-cysteine (Sigma-Aldrich #164739), prepared as a 100 mM stock solution in DMSO and stored at −20 °C, was applied to arrest cells at prometaphase.

### Preparation of Cell Homogenates

HEK cells were directly detached in medium using ice-cold 1xPBS, followed by two washes with ice-cold 1xPBS and collected by centrifugation (300*g*, 5 min at 4 °C). The cell pellet was resuspended in an appropriate amount of ice-cold NP-40 lysis buffer [50 mM TrisHCl, pH 7.4, 10% (v/v) Glycerol; 100 mM NaCl, 2 mM MgCl_2_, 1% (v/v) NP-40 with freshly added 1 mM DTT, 10 μg/ml aprotinin, 10 μg/ml leupeptin and 10 μg/ml Pefabloc]. Protein concentration was determined by Bradford Assay using BSA as a standard. Cell homogenates were mixed with 5× Laemmli sample buffer (Tris/HCl (pH 6.8) 0.1 M, EDTA 5 mM, SDS 15% (w/v), sucrose 40% (w/v), β-mercaptoethanol 10% (v/v), Bromophenol Blue 0.02%), to a final concentration of 1× sample buffer and boiled at 100 °C for 5 min. Prepared samples were stored at −20 °C.

### TurboID-dependent Biotinylation

TurboID proximity labeling was conducted as described by Cho *et al*. (29) with specific modifications. Approximately 20 million HeLa or HEK cells expressing a TurboID construct were seeded in T150 flasks and incubated in complete selective DMEM for 24 h (four biological replicates per experiment). For HeLa TurboID cells, 10 mM sodium-butyrate was added to the medium 16 h before biotin labeling. The medium was then replaced with warm complete DMEM supplemented with 50 μM freshly prepared biotin (Sigma-Aldrich #B4501; biotin stock was prepared as a 100 mM solution in DMSO and stored at −20 °C) to initiate labeling at 37 °C for 10 min. The 10-min labeling pulse was chosen based on prior optimization and published TurboID protocols, which demonstrated that short labeling windows maximize proximity specificity while minimizing nonspecific background (29). Negative controls without biotin were included for optimization.

After biotinylation, cells were washed five times on ice with ice-cold 1 × Dulbecco’s PBS (Thermo Fisher Scientific #21600010) and detached using cold Dulbecco’s PBS *via* pipetting (HEK cells) or scraping (HeLa cells). The cell suspension was collected and centrifuged at 300*g* for 5 min at 4 °C, and the supernatant was discarded. The cell pellet was resuspended and lysed in 1 ml RIPA lysis buffer (50 mM Tris, 150 mM NaCl, 0.1% SDS, 0.5% sodium deoxycholate, 1% Triton X-100 in ddH_2_O, pH 7.5), supplemented with a protease inhibitor cocktail (0.1 mg/ml Pefabloc, 0.01 mg/ml leupeptin, 0.02 U/ml aprotinin and 1 mM PMSF (prepared as a 100 mM solution in isopropanol, aliquoted, and stored at −20 °C), and incubated on ice for at least 10 min. Lysates were clarified by centrifugation at 16,600*g* for 10 min at 4 °C, and supernatants were transferred to new Eppendorf tubes. Protein concentration was determined using the Bradford Assay.

Approximately 3.2 mg of total protein, supplemented with an additional 500 μl RIPA lysis buffer, was incubated with 350 μl pre-equilibrated Streptavidin magnetic beads (Thermo Fisher Scientific #88817) for protein affinity purification. The mixture was rotated at room temperature for 1 h and then left rotating overnight at 4 °C. Aliquots of whole cell lysates (WCLs) that were not required for affinity purification were saved and stored at −20 °C. Beads were collected using a magnetic rack, and supernatants (flow-through) were stored at −20 °C.

Beads were subsequently washed twice with 1 M KCl (1 ml, 2 min at room temperature), once with 0.1 M Na_2_CO_3_ (1 ml, ∼10 s), twice with 2 M urea in 10 mM Tris-HCl (pH 8.0, prepared fresh), and twice with RIPA lysis buffer (1 ml per wash, 2 min at room temperature). For samples designated for mass spectrometry analysis, the final RIPA buffer washes were omitted, and the samples were further processed for mass spectrometry in the final urea wash buffer.

Biotinylated proteins were eluted from the beads by transferring them to fresh tubes and boiling in 30 μl 3× protein loading buffer supplemented with 2 mM biotin and 20 mM DTT at 95 °C for 10 min. 1 M DTT stock solution (Thermo Fisher Scientific #BP172-5) was prepared in dH_2_O and stored at −20 °C. The eluates were collected by removing the beads using the magnetic rack. Aliquots of WCL, FT, W1 and eluates were treated with 6× protein loading buffer (0.33 M Tris (pH 6.8), 34% (vol/vol) glycerol, 10% (wt/vol) SDS, 0.09% (wt/vol) DTT, 0.12% (wt/vol) bromophenol blue in ddH_2_O) and heated at 95 °C for 10 min before storage at −20 °C for Western blot analysis.

### Western Blotting

Protein samples were separated through sodium dodecyl sulfate polyacrylamide gel electrophoresis. The proteins were then electrophoretically transferred onto a Polyvinylidene Fluoride membrane (Roche, Sigma-Aldrich, catalog number 03010040001) overnight.

The membrane was blocked with 5% non-fat dry milk in TBS containing 0.05% Tween 20 (TBST) for 1 h at room temperature. To detect biotinylated proteins, the membrane was incubated with streptavidin–HRP (1:3000) in 3% BSA (wt/vol) in 1× TBST at room temperature for 30 min. Alternatively, for detecting the expression of endogenous proteins or overexpressed constructs, the membrane was incubated with primary antibodies in 3% BSA (wt/vol) in 1× TBST overnight at 4 °C.

Following primary antibody incubation, the membrane was washed three times with TBST, each wash lasting 5 min. Subsequently, it was subjected to incubation with the appropriate HRP-conjugated secondary antibody in 3% BSA (wt/vol) in 1× TBST for 1 h at room temperature. After three additional washes with TBST, the protein bands were visualized using Super Signal West Pico Substrate (Thermo Fisher Scientific, catalog number 34078) in accordance with the manufacturer's instructions. Signals were detected with a ChemiDoc MP Imaging System from Bio-Rad.

### Image Acquisition and Analysis

Confocal fluorescence microscopy images were recorded using a 63×/1.4 NA objective mounted on a spinning disk confocal laser-scanning microscope (UltraVIEW VoX, PerkinElmer; Eclipse Ti, Nikon). Z-stack sections of 0.5 μm, covering the entire depth of the cell, were acquired and total projections were calculated.

### LC-MS/MS and Data Analysis

Samples were analyzed using an EASY-nLC 1200 (Thermo Fisher Scientific) coupled to a Q Exactive HF or Exploris 480 mass spectrometer (Thermo Fisher Scientific), respectively. Peptides were separated and sprayed with 25 cm fused silica emitters (75 μm inner diameters, CoAnn Technologies) packed in-house with ReproSil-Pur 120 C_18_ AQ 1.9 μm (Dr Maisch). 0.5 μg of peptides per sample were analyzed using a stepped gradient of 0% to 45% solvent B (80% ACN, 0.1% FA) in 60 min at 250 μl/min, or 0% to 55% solvent B in 100 min at 300 μl/min, followed by wash steps. Peptide survey mass spectra were acquired in the Orbitrap analyzer, with a resolution of 120,000 on the Exploris 480 and 60,000 in MS^1^ for the Q Exactive HF. A resolution of 15,000 for MS^2^ spectra was used on both instruments. The scanned mass range was 300 to 1750 m/z. The normalized collision energy was set to 25. Peptides with a charge of +1, > +6, or peptides with an unassigned charge state were excluded from fragmentation.

Processing of raw data was performed using the MaxQuant software version 2.1.3.0 (https://www.maxquant.org/) with default settings (30) MS/MS spectra were assigned to the UniProt human reference proteome with 20,427 entries (31). During the search, sequences of 248 common contaminant proteins as well as decoy sequences were automatically added. Trypsin specificity was required and a maximum of two missed cleavages was allowed. Carbamidomethylation of cysteine residues was set as fixed, oxidation of methionine and protein N-terminal acetylation as variable modifications. A false discovery rate of 1% for peptide spectrum matches and proteins was applied. Match between runs, as well as LFQ and iBAQ (32, 33) were enabled. A tolerance of 20/4.5 ppm was allowed for first/main search, while the MS/MS peak tolerance was 20 ppm.

The MaxQuant protein groups table was imported in R and subsequent data processing steps were performed. All LC–MS/MS measurements were performed on independent biological replicates (separate cell cultures and TurboID labeling reactions). Specifically, LFQ intensities were log_2_ transformed to facilitate further analysis. To address missing values in the data, the impute.QRILC function from the imputeLCMD package (34) was employed. This imputation process was specifically applied to both experimental and plain TurboID control samples. High-confidence proximity interactors were defined by differential enrichment relative to a cytosolic TurboID-NES control, which subtracts nonspecific cytosolic biotinylation, but does not control for general outer mitochondrial membrane–wide background labeling. The utilization of imputation techniques is common in quantitative proteomics to handle missing values and ensure a comprehensive dataset for calculating fold-changes and statistical analysis. Protein groups were considered for further analysis if they were quantified in more than 2 replicates. For statistical evaluation of the differential enrichment analysis limma (version 3.46.0) (35) was used to compare LFQ intensities of controls and the specific TurboID fusion protein pulldowns. Resulting *p*-values were adjusted for multiple testing using the FDR, and proteins with log_2_ fold change ≥1 and adjusted *p*-value (FDR) < 0.05 were considered significantly enriched.

## Results and Discussion

### Characterization of Myo19, Miro2 and MTX3 TurboID Constructs

TurboID is a highly reactive biotin ligase, that upon fusion with proteins of interest enables rapid biotinylation of transient protein-protein interactions (26, 36). Biotinylated proteins are then identified by mass spectrometry to reveal protein-protein interaction networks efficiently.

**Fig. 1:**
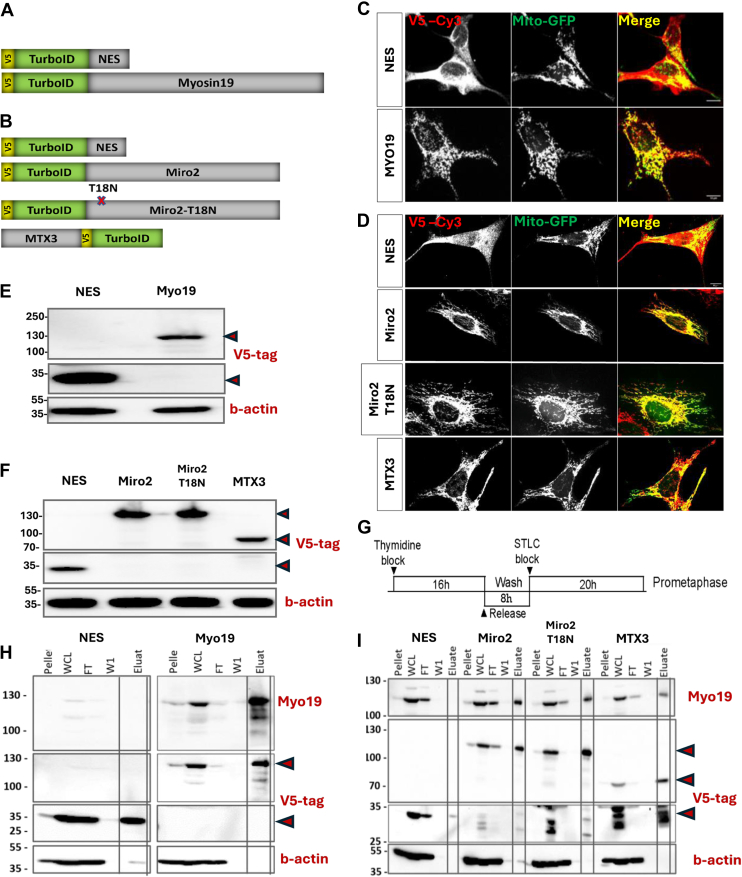
**Characterization of Myo19, Miro2 and MTX3 TurboID constructs.***A* and *B,* schematic representations of TurboID constructs used in the present study. The TurboID-Myo19 construct includes a V5-tag and TurboID fused to the N-terminus of full-length Myo19. A construct of V5-TurboID- NES served as a control (*A*). They were stably transfected into Myo19 KO HEK cells. In *B*) the TurboID-Miro2, TurboID-Miro2 T18N and MTX3-TurboID constructs are shown which were stably transfected into HeLa cells. *C* and *D,* confocal fluorescence microscopy images of Myo19-deficient HEK cells (*C*) and HeLa cells (*D*) stably expressing TurboID constructs. Mitochondria were visualized using Mito-EGFP (*green*) and TurboID constructs were stained with anti-V5 antibody (*red*). HeLa cells were treated with 10 mM sodium butyrate overnight to enhance protein expression. The scale bars represent, 10 μm. *E* and *F,* immunoblot analysis of expressed TurboID constructs. *E,* shows TurboID-Myo19 and TurboID-NES constructs in Myo19-deficient HEK cells, while (*F*) shows TurboID-Miro2, Miro2-T18N, MTX3, and NES constructs in HeLa cells. *Blots* were incubated with the indicated antibodies. β-actin served as a loading control. *G,* schematic illustration of applied timeline for cell cycle synchronization and arrest at prometaphase. *H* and *I,* verification of the selective biotinylation activity of TurboID constructs. Immunoblots of Myo19-deficient HEK cells (*H*) and HeLa cells (*I*) show self-biotinylation of the expressed TurboID constructs. Aliquots of nuclear pellet fractions (*Pellet*), whole cell lysates, flow-through, wash 1, and eluates from streptavidin beads were separated on sulfate polyacrylamide gel electrophoresis and immunoblotted with anti-V5, anti-Myo19 and anti-β-actin antibodies. *Arrowheads* indicate the respective V5-tagged constructs, showing approximate molecular weights of 130 kDa for TurboID-Myo19, 35 kDa for TurboID-NES, 110 kDa for both TurboID-Miro2 and TurboID-Miro2 T18N, and 75 kDa for MTX3-TurboID. NES, nuclear export sequence; MTX3, metaxin-3; Myo19, myosin-19 interphase.

To discover potential partners interacting with Myo19 in living cells, V5 epitope-tagged TurboID was fused to the N-terminus of full length Myo19 (Fig. 1*A*). We stably transfected Myo19-deficient HEK cells (17) with TurboID-Myo19. As a control served a TurboID construct carrying a NES (Fig. 1*A*). We verified the correct subcellular localization of the expressed TurboID-Myo19 construct by immunofluorescence microscopy using the V5-epitope (Fig. 1*C*). Cells were transfected with Mito-EGFP to label the mitochondrial matrix and stained for the V5-epitope to label TurboID-Myo19. As expected, TurboID-Myo19 co-localized with mitochondria, whereas TurboID-NES was detected in the cytosol. The expression of the TurboID constructs was subsequently confirmed by immunoblotting (Fig. 1*E*) using an anti-V5 epitope antibody, revealing the expected molecular masses of approximately 130 kDa for TurboID-Myo19 and 35 kDa for TurboID-NES.

To obtain more complete information on the Myo19 interaction network and that of its interaction partners Miro2 and MTX3, TurboID fusions were constructed with Miro2 (Rhot2), a Miro2 mutant and MTX3 (Fig. 1*B*). Since the interaction between Miro2 and Myo19 is dependent on the nucleotide state of the N-terminal GTPase domain of Miro2 (12, 14), threonine residue 18 was replaced with an asparagine residue yielding Miro2 T18N that is no longer able to bind GTP (Fig. 1*B*).

We established stably transfected HeLa cell clones expressing TurboID-Miro2, TurboID-Miro2 T18N, MTX3-TurboID and TurboID-NES at low levels. To enhance expression, cells were treated overnight with sodium butyrate (10 mM), a histone deacetylase (HDAC) inhibitor that increases histone acetylation and thereby promotes transcription from integrated constructs (37, 38). Mitochondrial localization of these TurboID-constructs was verified by immunofluorescence staining of the HeLa cells (Fig. 1*D*). TurboID-Miro2, TurboID-Miro2 T18N, and MTX3-TurboID co-localized with mitochondria, while TurboID-NES was localized in the cytosol. The expression of the correct TurboID fusion proteins was subsequently confirmed by immunoblotting (Fig. 1*F*). They showed the expected molecular masses of approximately 110 kDa for TurboID-Miro2 and TurboID-Miro2 T18N, and of 75 kDa for MTX3-TurboID.

Next, we analyzed the biotinylation of cellular proteins catalyzed by the TurboID constructs after the addition of 50 μM biotin for 10 min to the cells by immunoblotting of whole cell lysates with streptavidin-horseradish peroxidase conjugate (Supplemental Fig. S1, *A* and *B*). For comparison, controls were included that were not incubated with biotin to evaluate endogenously biotinylated proteins and the background levels of biotinylation. A set of specifically biotinylated proteins was observed for each TurboID fusion construct spanning a wide molecular weight range (Supplemental Fig. S1, *A* and *B*).

The V5-tagged TurboID constructs were biotinylating themselves as shown by affinity purification. Whole cell lysates generated from labelled cells were incubated with streptavidin-coated magnetic beads. They were washed thoroughly under denaturing conditions. Biotinylated proteins were eluted from the beads by boiling in SDS protein-loading buffer supplemented with free biotin and DTT. V5-tagged TurboID-Myo19 and TurboID-NES were successfully enriched in the respective eluates, whereas β-actin was not (Fig. 1, *H* and *I*). Effective biotinylation was also consistently achieved in HeLa WT cells expressing the other TurboID fusion proteins. In these eluates, we detected not only our bait proteins Miro2, Miro2 T18N or MTX3, but also specifically Myo19 (Fig. 1*I*). This co-elution of Myo19 confirmed its proximity to Miro2 and MTX3. Subsequently, mass spectrometry was employed to identify biotinylated proteins isolated by streptavidin affinity capture.

In previous work, we demonstrated that in the absence of Myo19, unlike in WT cells, mitochondrial fusion was not inhibited at prometaphase (17). Furthermore, in the absence of Myo19 mitochondria accumulated during mitosis asymmetrically at the spindle poles. To define the proximity networks for Myo19 during interphase and prometaphase, we synchronized and arrested cells in prometaphase (PM) as schematically depicted in Figure 1*G*, before the start of the proximity labelling.

### Proximity Interaction Networks of Myo19, Miro2, Miro2 T18N and MTX3

Affinity purified biotinylated proteins were analyzed by mass spectrometry. Identified proteins were considered high confidence proximity interactors when they showed a minimum twofold enrichment compared to the control group and had an adjusted *p*-value FDR ≤ 0.05 based on LFQ intensities and limma statistical analyses. Our investigation unveiled 560 high-confidence Myo19 proximity interactors during interphase and 272 during prometaphase (jPOST JPST004008). Of those proximity interactors, 52 were identified both at interphase and prometaphase (jPOST JPST004008). As specific members of the Miro2 proximity network we identified 342 proteins (jPOST JPST004008). For the mutant Miro2 T18N a reduced number of 200 proteins satisfied the specificity criteria (jPOST JPST004008). 50 proteins were common between Miro2 and Miro2 T18N. A similar number of 216 proteins were identified as being in the proximity of MTX3 (jPOST JPST004008). Because TurboID-Myo19 proximity labeling was performed in Myo19-KO HEK cells, whereas TurboID–Miro2 and MTX3 experiments were performed in WT HeLa cells, some of the noted differences between the proximity networks of Myo19 and the other proteins might be due to cell-type in addition to bait-specific effects. Enrichment was assessed relative to a cytosolic TurboID-NES control and did not include additional unrelated outer mitochondrial membrane proteins.

**Fig. 2:**
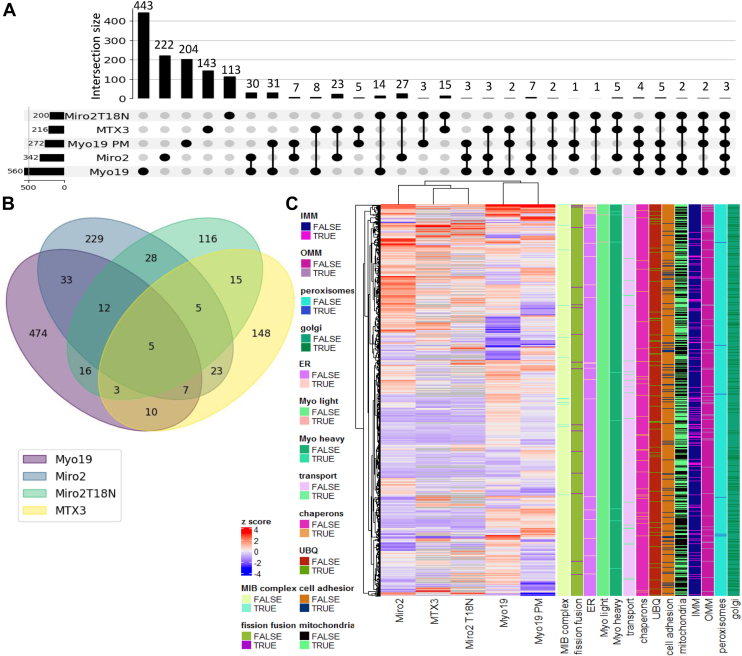
**Comparative analysis of TurboID proximity labeling for Myo19 in interphase and prometaphase, Miro2, Miro2 T18N and MTX3.***A,* UpSet plot illustrating the distribution of differentially enriched proteins across the five indicated TurboID screens (log_2_ fold change ≥1 and false discovery rate-adjusted *p*-value ≤0.05). *B,* Venn diagram showing the overlap of significantly enriched proteins among Myo19, MTX3, Miro2, and Miro2 T18N. *C*, Heatmap of Z-score normalized logFCs for protein groups across Myo19 (interphase and prometaphase), Miro2, MTX3, and Miro2 T18N, with functional classifications indicated by *color bars*. Heatmap *colors* represent Z-scores of log_2_ fold changes, with *gray* indicating missing data (NA). Data are based on n = 4 independent biological replicates per group. Created with ComplexHeatmap (79). MTX3, metaxin-3; Myo19, myosin-19 interphase; Myo19 PM, myosin-19 prometaphase.

However, all of our described TurboID screens used outer mitochondrial membrane proteins facing the cytosol as baits. This allowed us to develop a more comprehensive nearest neighbor analysis, keeping in mind that outer mitochondrial membrane proteins can diffuse within the plane of the membrane. We conducted a differential enrichment analysis between the TurboID screens of Myo19, Miro2, Miro2 T18N and MTX3. To this end, we generated an upSet plot and a Venn diagram that highlight differentially enriched proteins (Fig. 2, *A* and *B* and jPOST JPST004008).

The results revealed that five proteins (MAVS, USP33, MTFR1L, EXD2 and VPS13D) were common in all of the four interphase screens. All of those proteins have been reported to be associated with the outer mitochondrial membrane (39, 40, 41, 42, 43, 44, 45). Six more proteins, all of them outer mitochondrial membrane proteins (MTX2, SNAP29, SNAP47, BCL2L13, BNIP3, and CISD1), were part of the Myo19, Miro2 and MTX3 proximity networks (jPOST JPST004008 and Fig. 2, *A* and *B*). Additionally, 23 proteins were identified as mutually enriched between Miro2 and MTX3, but not Myo19 or Miro2 T18N. Notably, proteins enriched across multiple baits relative to the cytosolic TurboID-NES control may partly reflect general OMM residents rather than strictly bait-specific proximity partners, an inherent limitation of proximity labeling within the same membrane compartment.

We generated a heat map based on Z-score normalized fold-changes to visually represent the relation between enriched protein groups in Myo19 interphase (INT), Myo19 PM, Miro2, Miro2 T18N and MTX3. This heat map utilized standardized Z-scores derived from log_2_-fold changes in LFQ (Fig. 2*C*). The heat map not only illustrates the relative abundance of proteins across the samples but also provides insights into the involvement in the mitochondrial intermembrane bridging (MIB) complex, endoplasmic reticulum-mitochondria contact sites (ERMCS) and the process of fusion/fission.

**Fig. 3:**
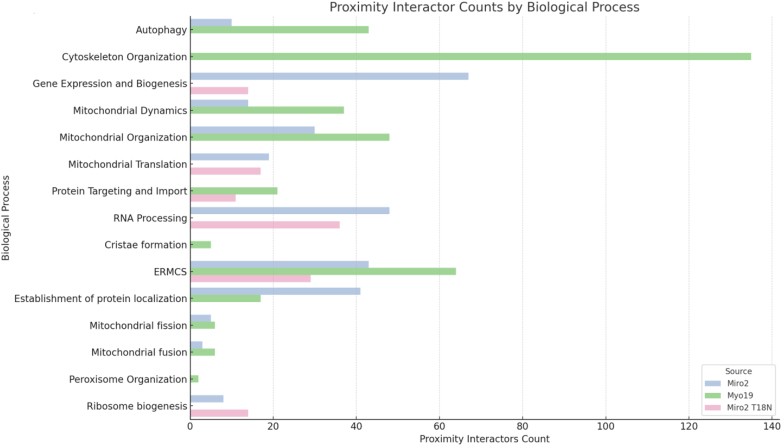
**Comparative Analysis of Biological Processes Associated with the Proximity Networks of Myo19 Interphase, Miro2 and mutant Miro2 T18N.** For visualization Google Colaboratory was used. Colors of the individual screens are indicated.

In accordance with previous reports that ribosomes associate with the outer mitochondrial membrane (46, 47, 48), we found in the proximity networks of Myo19, Miro2 and MTX3 a substantial number of proteins that play a role in translation, RNA processing and ribosomal biogenesis (Fig. 3*A*, jPOST JPST004008).

Based on previous work, we expected to find proteins such as CENPF, TRAK1/2 and MTFR2 only in the proximity network of Miro2, but not in Miro2 T18N (11, 49). This was indeed the case (jPOST JPST0040081). We observed that ARMC1 was exclusively associated with the proximity network of Miro2 and not with Miro2 T18N. ARMC1 is a member of the Armadillo (Arm)-repeat protein family. It is related to ArmCx proteins, which play a role in mitochondrial dynamics. A study by Wagner *et al*. (50) reported that ARMC1 interacts with various components of the MICOS/MIB complex and MTFR1L, a regulator of mitochondrial fission. Common to both proximity networks of Miro2 and Miro2 T18N were 40 proteins, among them Myo19, MTFR1 and MTFR1L. This was contrary to the expectation that the mutation abrogates binding of Myo19, MTFR1 and MTFR1L to Miro2. However, Myo19 was more enriched in Miro2 (log_2_ fold change = 8.5) than in Miro2 T18N (log_2_ fold change = 5). Similarly, MTFR1 and MTFR1L showed greater enrichment in Miro2 (log_2_ fold change = 7.8 and 7.11, respectively) than in Miro2 T18N (log_2_ fold change = 5 and 4.69, respectively). Miro has been reported to dimerize and multimerize (51, 52). As we expressed these two Miro2 TurboID constructs in WT cells that express endogenous Miro1/2, this may explain the residual proximity of Myo19 to Miro2 T18N. However, an alternative explanation for the proximity of Myo19 to Miro2 T18N might be that Myo19 also associates with the outer mitochondrial membrane independently of Miro1/2 by binding to lipids and MTX3 (21, 23, 53, 54, 55). Although several mitochondrial dynamics proteins (including Myo19 MTFR1, MTFR1L, RHOT2 and MAVS) remain detectable in the Miro2-T18N proximity network, volcano-plot analyses and direct Miro2 versus Miro2-T18N comparisons show that their enrichment is significantly reduced relative to WT Miro2, indicating a graded rather than all-or-none dependence on Miro2 GTP binding (Supplemental Figs. S2–S4). Based on functional enrichment analysis, the proximity network of Miro2 T18N was significantly depleted for proteins involved in mitochondrial dynamics, such as fusion, fission, organization and autophagy (jPOST JPST004008; Fig. 3).

### The Proximity Networks of Myo19 in Interphase and in Prometaphase

**Fig. 4:**
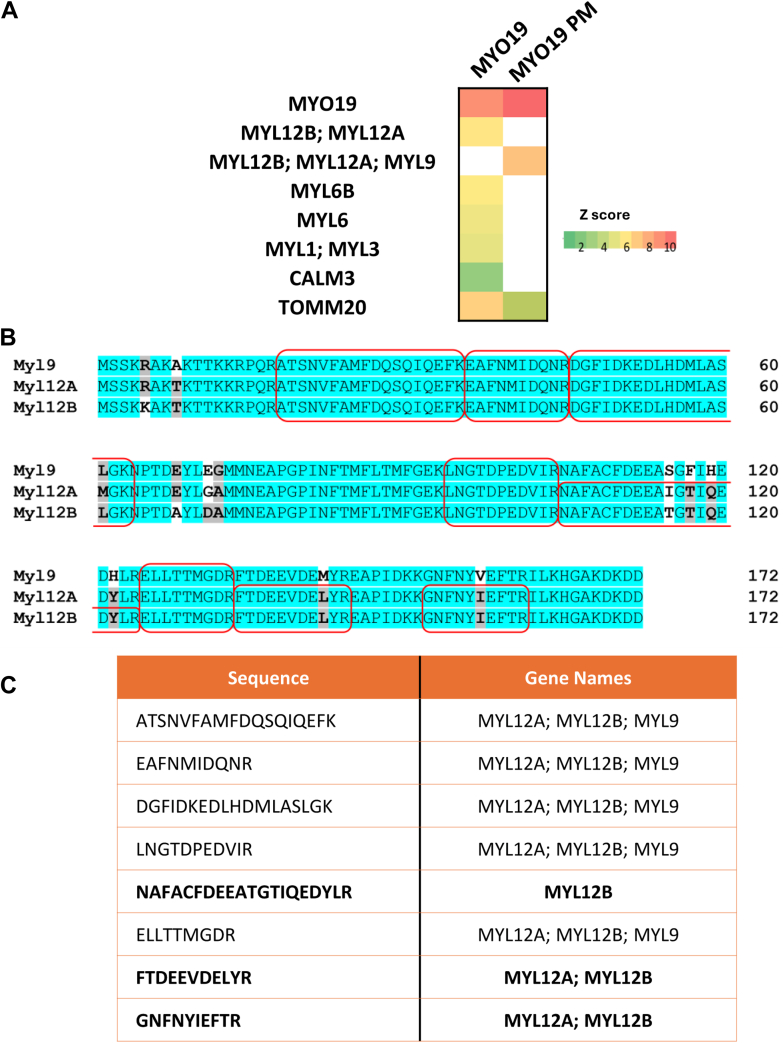
**Enrichment of Myosin Light Chains in Myo19 TurboID Screens.***A,* heatmap of enriched myosin light chains at interphase (Myo19) and at prometaphase (Myo19 PM) of Myo19 TurboID screens based on label-free quantification. *B,* amino acid alignment for regulatory light chains MYL9, MYL12A and MYL12B. Peptides identified in the TurboID screens are boxed. *C,* sequences of identified peptides for regulatory light chains Myl12A, Myl12B and Myl9 in the Myo19 TurboID screens.

Myo19 contains three IQ-motifs representing putative binding sites for myosin light chains. Based on structural information, the N-terminus of Myo19 to which the Turbo biotin ligase was fused, is predicted to be positioned close to the light chain binding region (56). Therefore, it appears likely that the light chains will be biotinylated both in interphase and at prometaphase. The only myosin light chain that was detected both in interphase and prometaphase was the regulatory light chain Myl12B (Fig. 4, *A*–*C*). The essential light chains Myl6B, Myl6, Myl1/3 and calmodulin were detected with different abundance only in interphase, but not prometaphase (Fig. 4*A*). Therefore, the final assignment of Myo19 light chains, except for Myl12B, remains a matter of debate (57, 58). We noted a significant enrichment of Tomm20 in both the interphase and prometaphase Myo19 TurboID screens (Fig. 4*A*), similarly to its reported binding partner AIP (59). Tomm20 recognizes and binds mitochondrial targeting sequences. These sequences share characteristics with IQ-motifs. Both form an amphipathic alpha-helix with a hydrophobic and a positively charged face. Therefore, Tomm20 might be able to interact with IQ-motifs and serve as temporary “light chain” mimetic. Further comprehensive biochemical and functional assays will be necessary to address this intriguing possibility. In this context, it is worth noting that deletion of Myo19 disturbs cristae organization.

The chaperone Unc45A that contributes to the folding of the myosin head domain (60, 61) was part of the Myo19 proximity network independent of the cell cycle phase. This was also true for the chaperones and dynein function regulatory proteins NudC, NudCD2 and NudCD3, but not NudCD1 (62). It will be interesting to determine the mechanism of their involvement in mitochondrial trafficking (63). In contrast, dynactin subunit 3 and cytoplasmic dynein 1 intermediate chain 2 of the microtubule minus end directed dynein/dynactin motor complex were part of the Myo19 prometaphase proximity network only, but not the interphase proximity network. Myo19 might coordinate transport of mitochondria with dynein/dynactin more closely during mitosis.

As a general Myo19 proximity network protein we detected in interphase and in prometaphase Armcx3 (jPOST JPST004008). This protein has been reported to represent an interaction partner of Miro and to regulate mitochondrial dynamics and trafficking (64). Therefore, Armcx3 might be involved in coordinating actin- and microtubule-based mitochondrial trafficking.

Myo19 was suggested to tether mitochondria to actin cables during mitosis (19). However, we did not find actin-associated proteins in the prometaphase proximity network. By contrast, during interphase we found a number of actin-associated proteins in the proximity of Myo19, such as gelsolin, tropomyosin isoforms TPM3 and TPM4/2, actin-related protein 2/3 complex subunit 5-like protein and tropomodulin 3 (jPOST JPST004008).

### Myo19-ERMCS Interaction Network in Interphase and Prometaphase

**Fig. 5:**
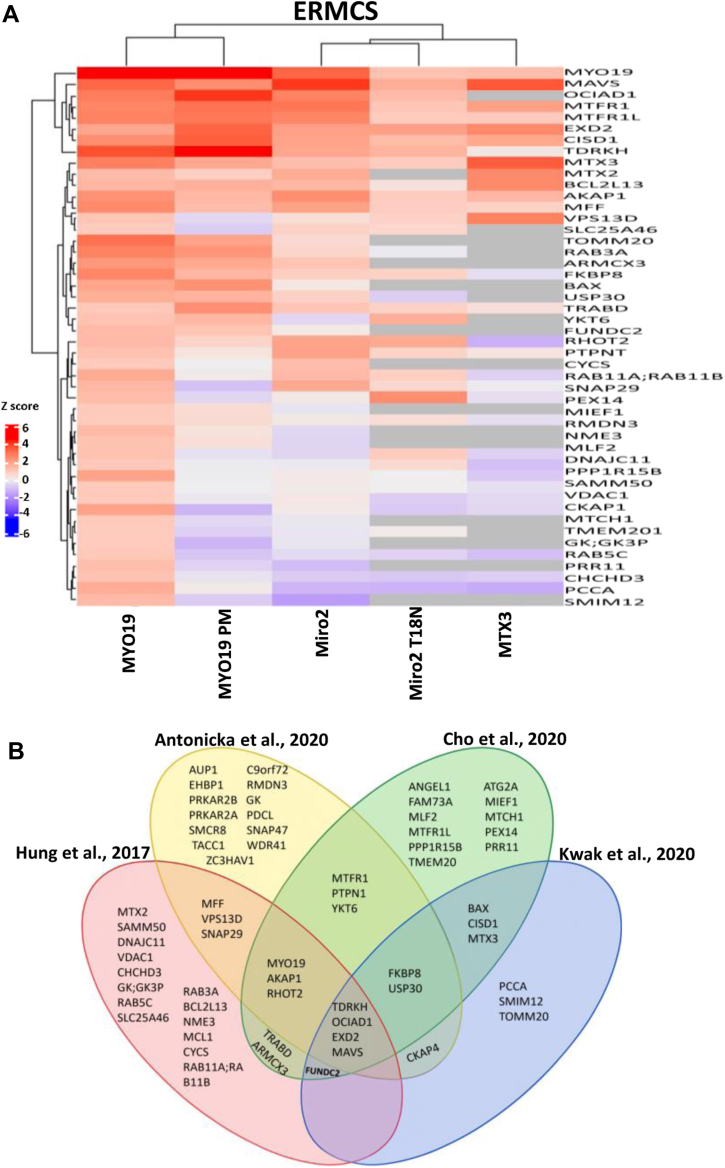
**Proteins of ERMCS enriched in Myo19 proximity networks.***A,* Heatmap of ERMCS proteins (GO:0044233 – ER–mitochondrion membrane contact site annotation) present in Myo19 (interphase) and Myo19 PM (prometaphase), Miro2, Miro2 T18N, and MTX3 networks. The heatmap displays the differential abundance of ERMCS-associated proteins, highlighting both unique and shared components of the endoplasmic reticulum–mitochondria contact sites detected in the indicated TurboID screens. *B,* Venn diagram of proteins identified in our Myo19 interphase TurboID screen overlapping with proteins, that were annotated as ERMCS proteins in four recently published proteomic studies (29, 65, 66, 67). See MSViewer *tnuwnnjdnj* for counts. ERMCS, ER-Mitochondria Contact Sites; MTX3, metaxin-3; Myo19, myosin-19.

Previous proteomic studies have classified Myo19 and Miro as proteins associated with endoplasmic reticulum-mitochondria contact sites (ERMCS) (29, 65, 66, 67). In agreement with these studies, we found that in Myo19-deficient HEK and mouse embryonic fibroblast cells, the number of ERMCS was reduced (22). Therefore, we examined the TurboID data for overlap with the reported ERMCS candidate proteome (Fig. 5*A*) and conducted a comparative analysis with four recent proteomic studies (29, 65, 66, 67) (Fig. 5*B*). The respective counts of reported ERMCS constituents found in our TurboID screens for Myo19 interphase (INT), Myo19 prometaphase (PM), Miro2, Miro2 T18N and MTX3 were 64, 22, 43, 29 and 15, respectively, emphasizing the classification of Myo19 as an ERMCS protein (Table 1).

**Table 1:** Comparison of TurboID candidate proteomes for Myo19 interphase, Myo19 prometaphase, Miro2, Miro2 T18N and MTX3 with published ERMCS proteomes

ERMCS proteomes	Hung et al., 2017	Kwak et al., 2020	Cho et al., 2020	Antonicka et al., 2020	ERMCS proteins
Technique used	APEX	Contact-ID	Split-ID	BioID	
ERMCS proteins	69	115	101	72	
Myo19-int	27	14	29	29	64
Myo19-pm	10	9	17	11	22
Miro2	17	18	21	21	43
Miro2 T18N	8	12	17	16	29
MTX3	9	7	7	6	15

ERMC, ER-Mitochondria Contact Sites.

The differences in the number of ERMCS proteins among the proximity networks of Myo19, Miro2 and Miro2 T18N might suggest distinct roles or levels of involvement in these structures for each protein variant. The high count of 64 ERMCS proteins in proximity of Myo19 during interphase confirmed its classification as an ERMCS protein. The Miro2 proximity network with 43 ERMCS proteins demonstrated a substantial, but comparatively more limited engagement with these structures in comparison to Myo19. Moreover, the count of 29 ERMCS proteins for Miro2 T18N suggests that the T18N mutation has a negative impact on Miro2's proximity to ERMCS. The least number of ERMCS proteins were found in the MTX3 proximity network. This finding indicated that the proximity of MTX3 to ERMCS is more limited and that it might have alternative functions. This finding is consistent with a recent analysis of ERMCS in MTX3 KO cells (23).

Further, our data showed that Myo19 and ERMCS are regulated by the cell cycle. The Myo19 prometaphase ERMCS proximity network contained only one third of the proteins found in interphase. However, Mitoguardin 1 (Fam73A) that in analogy to Mitoguardin 2 (Fam73B) might be part of ERMCS and serve as a lipid transfer protein (68, 69) was identified in both Myo19 proximity networks. The ER protein inositol 1,4,5-trisphosphate receptor subtype 1 was in proximity of Myo19 only in the prometaphase, but not the interphase. This agrees with the recent reports that ERMCS expand during mitosis and Ca^2+^ transfer is enhanced stimulating ATP production (70, 71, 72).

### Myo19 is Part of the MIB/MICOS Network

**Fig. 6:**
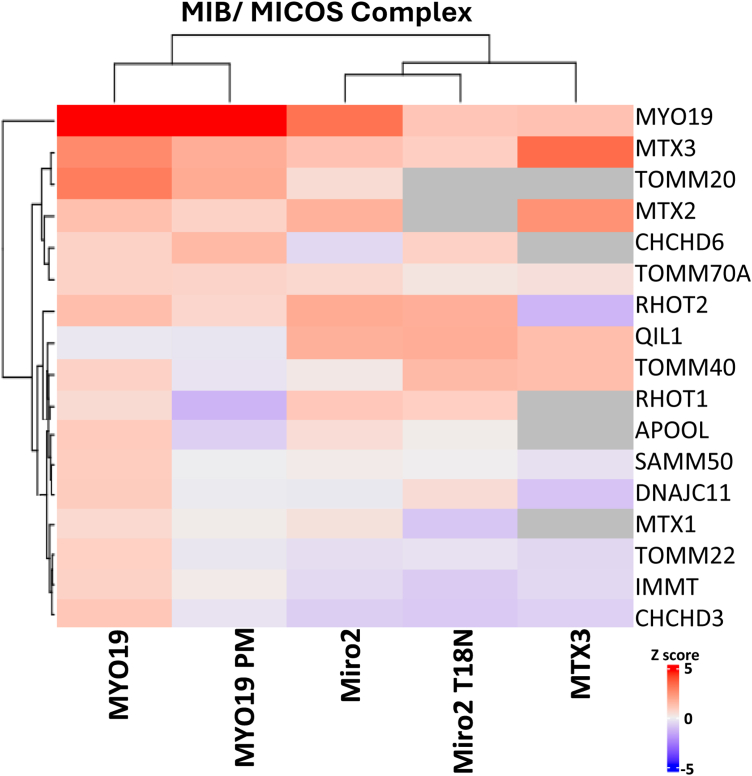
**MIB/ mitochondrial contact site and cristae organizing system proteins enriched in the indicated TurboID screens.** Heatmap showing proteins of the MIB/ mitochondrial contact site and cristae organizing system complex with Z-score normalized log_2_ fold changes. PM indicates enrichment in prometaphase.

Proteins of the MIB/MICOS complex regulate cristae morphology (73, 74). In our previous work, we showed that cristae morphology is altered in both Myo19-deficient HEK and mouse embryonic fibroblasts (22). Additionally, these cells had compromised mitochondrial respiratory profiles and reduced energy production. A protein complex known as MICOS that is anchored in the inner mitochondrial membrane, controls cristae organization (74). This complex in turn is associated with the sorting and assembly machinery (SAM) complex anchored in the outer mitochondrial membrane. Together they form the MIB that connects the IMM with the OMM (75, 76). Myo19 has been suggested to interact with metaxin-3, which is considered to be part of the SAMM50 complex (12, 21, 23, 24, 54). In the TurboID screens and especially in those with interphase Myo19, many of the SAM/MIB components were enriched (Fig. 6). However, the only MIB-complex protein that was enriched in all of the Myo19, Miro2 and MTX3 screens was MTX2. Most of the MIB/MICOS complex proteins were part exclusively and significantly of the Myo19 interphase proximity network. These proteins included CHCHD3/Mic 19, APOOL/Mic 27, DNAJC11, SAMM50 and MTX3. To label the Mic 19 and 27 proteins localized in the intermembrane space, the short-lived biotin intermediates must have crossed the outer membrane, or the proteins were biotinylated during their import. Similar, to the reduced number of ERMCS proteins found in proximity of Myo19 at prometaphase, also the number of MIB/MICOS components in proximity of Myo19 was greatly reduced at prometaphase in comparison to the interphase. This finding indicates that the proximity of Myo19 to ERMCS and MIB/MICOS components is negatively regulated during mitosis.

MTX3 was reported to represent a component of the MIB complex (76). However, of the known core MIB components only MTX2 was identified as a strong hit in our MTX3 TurboID screen. MTX1 was not detected just as DNAJC11. SAMM50 was only a weak hit. Overall, the proximity network of MTX3 contained fewer proteins that had been assigned to the MIB/MICOS complex than Myo19 (Fig. 6). Although metaxins are involved in mitochondrial motility in *C. elegans* and mammalian neurons (77), no proteins engaged in microtubule-based transport could be identified in our MTX3 screen. The proximity network defined in the present study will serve as an important resource for future functional studies of MTX3.

The datasets presented here reflect spatial proximity of the identified proteins to the TurboID fusion proteins rather than direct physical interaction. The detection of proteins that are in proximity of the fusion proteins depends on labeling geometry, lysine accessibility and peptide detectability. Identified proximity partners were defined relative to a cytosolic TurboID-NES control, which enables robust comparative analyses across conditions of the four proteins examined that are all associated with the cytosolic face of the outer mitochondrial membrane. However, the proximity labeling of Myo19 and Miro2/MTX3 was performed in different cell types (HEK versus HeLa) which should be considered when interpreting quantitative differences across baits. This primarily affects absolute interactome definition, whereas the comparative and state-dependent analyses that form the core of this study rely on internally controlled datasets processed in parallel. While TurboID provides quantitative proximity information, orthogonal validation of selected candidates (*e.g.,* MTFR1L, ARMCX3) by coimmunoprecipitation or reciprocal proximity labeling will be important to confirm specific interactions.

In summary, this study defines the *in vivo* proximity networks of Myo19 during interphase and prometaphase, as well as the proximity networks of Miro2, Miro2 T18N, and MTX3. Comparative analysis revealed dynamic proximity networks regulated by cell cycle progression and Miro2 GTP binding. It places Myo19, Miro and MTX3 within the context of mitochondrial function and organization. Together, these datasets provide a comprehensive resource of information and offer multiple entry points for future functional studies.

## Data Availability

Raw data and MaxQuant output tables were uploaded to the jPOST Repository (78) and will be available under the identifier JPST004008.

Peak lists for the respective experiments were uploaded to MSViewer and can be viewed here:

**Table:** 

Comparison	Search Key
HEK KO TurboID Myo19 “INT” vs HEK KO TurboID NES “INT”	tnuwnnjdnj
HeLa TurboID Miro2 T18N vs HeLa TurboID NES	w02ytotpoe
HeLa TurboID Miro2 vs HeLa TurboID Miro2 T18N	fud6uwil1o
HeLa TurboID MTX3 vs HeLa TurboID NES	75jhydsnlq
HEK KO TurboID Myo19 “PM” vs HEK KO TurboID NES “PM”	v8iisdrrua
HeLa TurboID Miro2 vs HeLa TurboID NES	w02ytotpoe

## Supplemental data

This article contains supplemental data.
