# Supplementary material for: Dynamic Proximity Networks of Myosin-19 (Myo19) and its Mitochondrial Receptors Miro2 and Metaxin-3
Source: Mol Cell Proteomics. 2026 May 6;25(6):101582. doi: 10.1016/j.mcpro.2026.101582 (PMC13255069; doi:10.1016/j.mcpro.2026.101582)
Supplement: Supplementary figures [file mmc1.pdf]

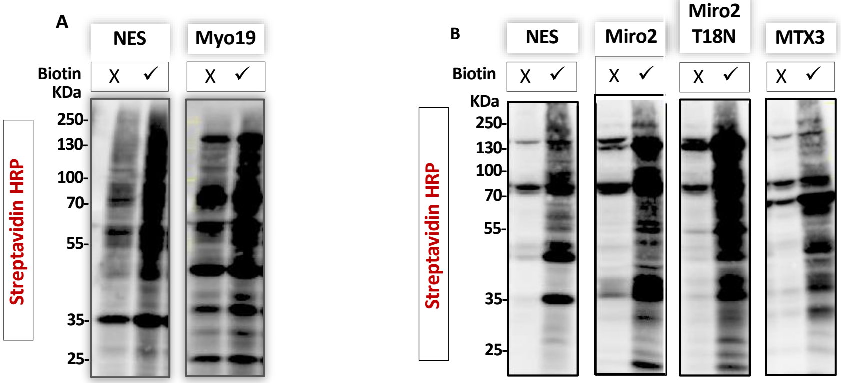

Supp. Fig. S1: Characterization of protein biotinylation induced by TurboID constructs. A) Myo19-deficient HEK cells stably expressing TurboID-Myo19 or TurboID-NES. B) Hela WT cells stably expressing TurboID-Miro2, TurboID-Miro2T18N, MTX3-TurboID and TurboID-NES. Cells were treated with (?) and without (X) 50 µM biotin for 10 min and then lysed. Whole-cell lysates were separated by SDS-PAGE and analyzed by streptavidin–HRP blotting.

<u>**Miro2 –TurboID vs TurboID- NES**</u>

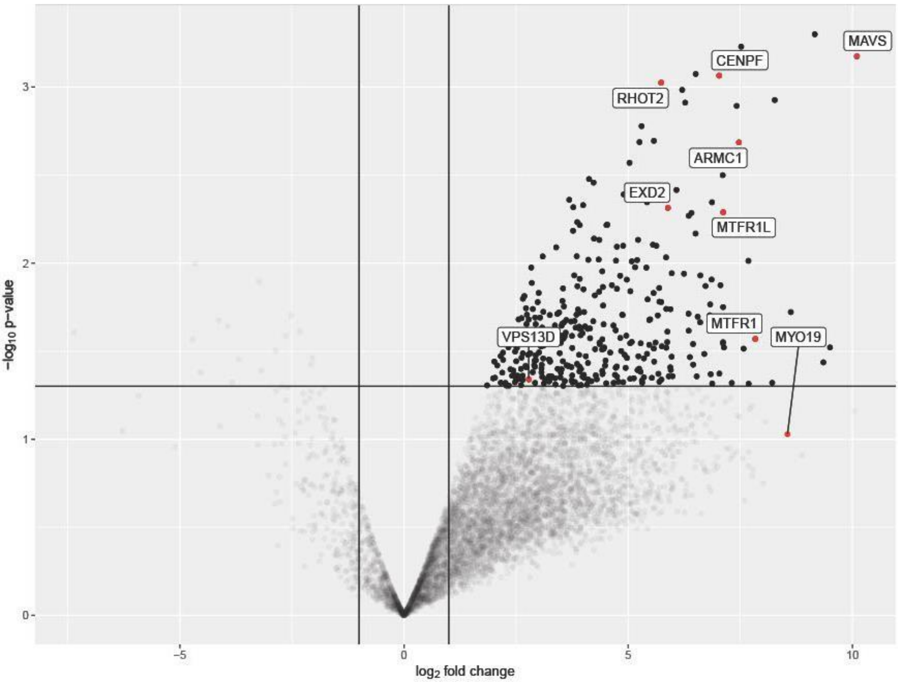

Supp. Fig. S2: Volcano plot of Miro2 TurboID versus TurboID-NES Volcano plot showing differential protein enrichment in the Miro2 TurboID proximity labeling experiment compared with the cytosolic TurboID-NES control. The x-axis represents $\log_2$ fold change (Miro2 TurboID / TurboID-NES), and the y-axis represents $-\log_{10}$ adjusted *p*-values. Proteins significantly enriched in the Miro2 proximity network were defined using a threshold of $\log_2$ fold change ≥ 1 and FDR-adjusted $p \leq 0.05$ (limma; $n = 4$ independent biological replicates). Selected known Miro2-associated and mitochondrial outer membrane proteins are highlighted.

Miro2 T18N–TurboID vs TurboID- NES

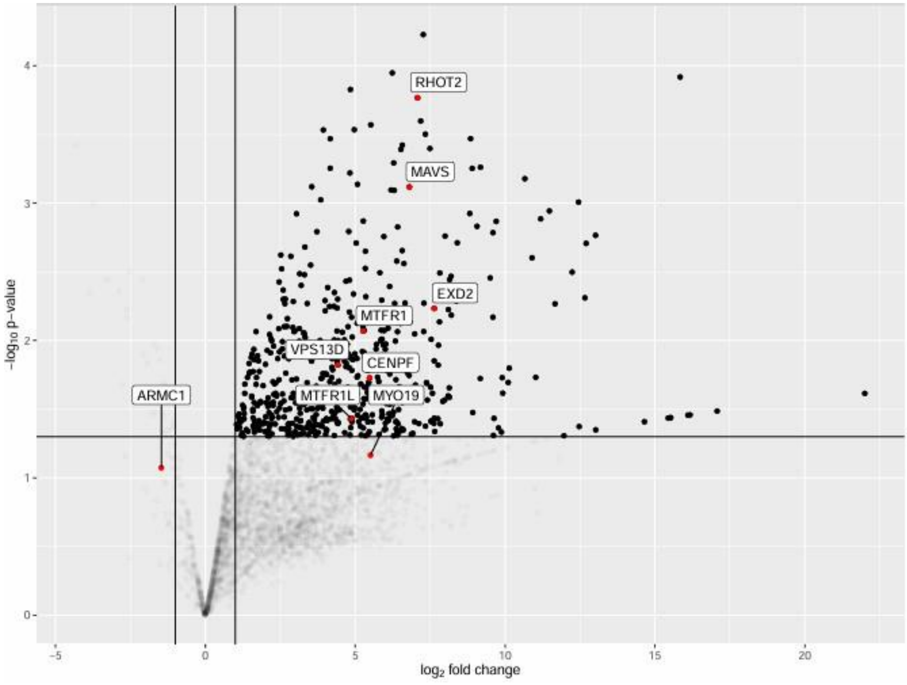

Supp. Fig. S3: Volcano plot of Miro2-T18N TurboID versus TurboID- NES.
Volcano plot showing differential protein enrichment in the proximity labeling experiment using the GTP-binding–deficient Miro2-T18N TurboID construct compared with the cytosolic TurboID-NES control. The x-axis represents $\log_2$ fold change (Miro2-T18N TurboID / TurboID-NES), and the y-axis represents $-\log_{10}$ adjusted *p*-values. Significantly enriched proteins were defined as $\log_2$ fold change ≥ 1 with FDR-adjusted $p \leq 0.05$ (limma; $n = 4$ independent biological replicates).

Miro2 –TurboID vs Miro2 T18N-TurboID

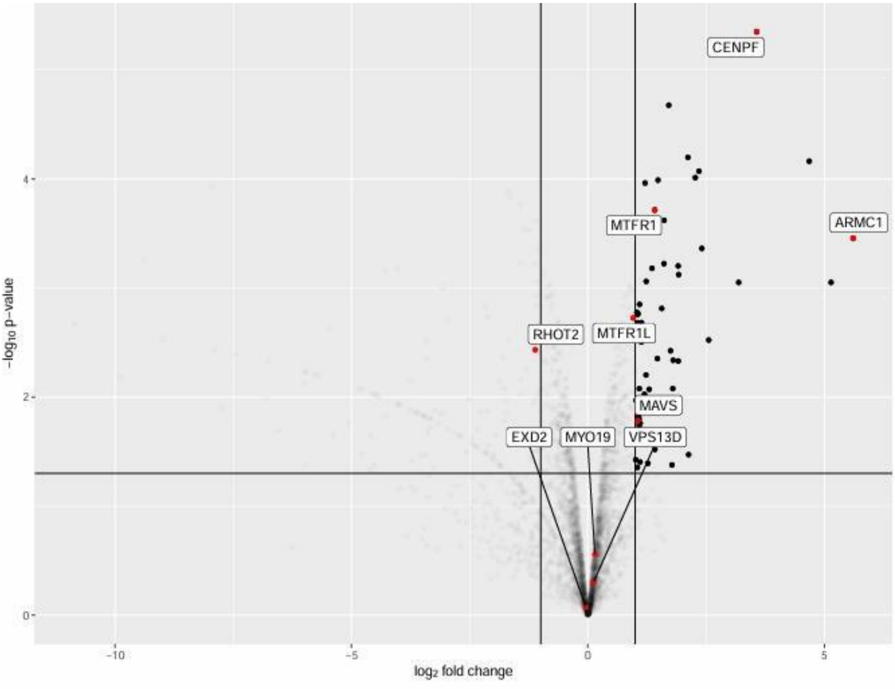

Supp. Fig. S4: Direct comparison of Miro2 and Miro2-T18N TurboID proximity networks.

Volcano plot showing differential protein enrichment between Miro2 TurboID and Miro2-T18N TurboID proximity labeling experiments. The x-axis indicates $\log_2$ fold change calculated as Miro2 TurboID relative to Miro2-T18N TurboID, while the y-axis shows $-\log_{10}$ FDR-adjusted p-values. Proteins significantly enriched between conditions were defined using a threshold of $|\log_2$ fold change$| \geq 1$ and FDR-adjusted $p \leq 0.05$ (limma; n = 4 independent biological replicates). Proteins with positive $\log_2$ fold change values are preferentially enriched in the Miro2 proximity network, whereas proteins with negative $\log_2$ fold change values are relatively enriched in the Miro2-T18N condition, indicating GTP-dependent modulation of Miro2-associated interactions.
